# A catalyst-free growth of aluminum-doped ZnO nanorods by thermal evaporation

**DOI:** 10.1186/1556-276X-9-256

**Published:** 2014-05-23

**Authors:** Syahida Suhaimi, Samsudi Sakrani, Tashi Dorji, Abdul Khamim Ismail

**Affiliations:** 1 Department of Physics, Faculty of Science, UTM, Skudai, Johor 81310, Malaysia; 2 Ibnu Sina Institute, UTM, Skudai, Johor 81310, Malaysia

**Keywords:** Al:ZnO nanowires, Thermal evaporation, Catalyst-free

## Abstract

The growth of Al:ZnO nanorods on a silicon substrate using a low-temperature thermal evaporation method is reported. The samples were fabricated within a horizontal quartz tube under controlled supply of O_2_ gas where Zn and Al powders were previously mixed and heated at 700°C. This allows the reactant vapors to deposit onto the substrate placed vertically above the source materials. Both the undoped and doped samples were characterized using scanning electron microscopy (SEM), field emission scanning electron microscopy (FESEM), energy-dispersive X-ray spectroscopy (EDX), high-resolution transmission electron microscopy (HRTEM) and photoluminescence (PL) measurements. It was observed that randomly oriented nanowires were formed with varying nanostructures as the dopant concentrations were increased from 0.6 at.% to 11.3 at.% with the appearance of ‘pencil-like’ shape at 2.4 at.%, measuring between 260 to 350 nm and 720 nm in diameter and length, respectively. The HRTEM images revealed nanorods fringes of 0.46 nm wide, an equivalent to the lattice constant of ZnO and correspond to the (0001) fringes with regard to the growth direction. The as-prepared Al:ZnO samples exhibited a strong UV emission band located at approximately 389 nm (*E*_*g*_ = 3.19 eV) with multiple other low intensity peaks appeared at wavelengths greater than 400 nm contributed by oxygen vacancies. The results showed the importance of Al doping that played an important role on the morphology and optical properties of ZnO nanostructures. This may led to potential nanodevices in sensor and biological applications.

## Background

Zinc oxide (ZnO) is an interesting and a well-known wide band gap II-VI semiconductor with a direct band gap of approximately 3.3 eV with large exciton binding energy (60 eV). The immense excitement in this area of research arises from understanding the fact that ZnO gives rise to new phenomena and multifunctionality which ultimately leads to unprecedented integration density with nanometer-scale structures [1]. Since the structural, optical, magnetic and electrical properties of ZnO are dependent on growth parameters, hence their applications. So, the prime interest here is to synthesize catalyst-free doped ZnO and learn the influence of dopant concentrations on the structural and optical properties. Over the time, researchers have used various dopants to dope ZnO NSs.

Doping semiconductor NWs with foreign elements to manipulate their electrical and magnetic properties is an important aspect for the realization of various types of advanced nanodevices [2]. Aluminum (Al) is one dopant that can be used to enhance phonon scattering promoted by Al induced grain reinforcement. The conductivity of the doped NWs is also increased.

## Methods

### Materials, method, and instruments

**Figure 1:**
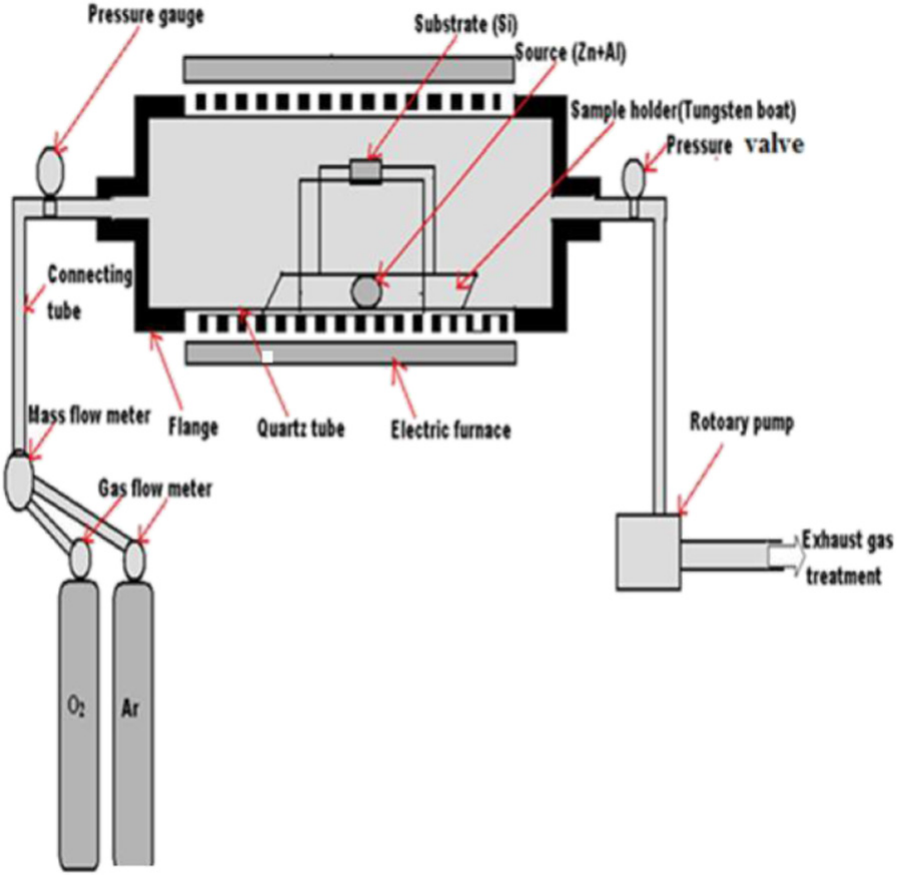
Schematic experimental setup for synthesis of ZnO:Al.

High purity Zn (99.99%), Al (99.7%), and oxygen (99.8%) were chosen as the source material. Silicon is used as a substrate and it must be cleaned to avoid the presence of contamination and impurity. Si slices were put in a beaker and cleaned in an ultrasonic bath for 30 min at temperature set 40°C with acetone and distilled water. Finally, the substrate is dried off with the aid of freeze dryer and stored in a desiccator. At temperature about 500°C, Zn would vaporize and get oxidized to ZnO by oxygen. The presence of a small amount of Al is expected to act as the dopant during the ZnO NSs growth which is expected to form ZnO:Al ultimately. A cleaned substrate (Si) was placed vertically above the sample holder as shown in Figure 1. Calculated and weighed mixture (Zn and Al) of 0.5 g was placed onto the substrate holder, and the setup was then loaded into the quartz tube carefully so that it is positioned at the center of the furnace/quartz tube. With the help of rotary pump attached to the furnace, tube chamber was initially evacuated to approximately 1 × 10^-2^ Torr pressure. This was important to remove undesirable gases which could be present initially. At a reduced pressure, it was also possible to achieve the temperature very quickly. With the programmable temperature controller, temperature of the oven was set to desirable value of 700°C.

The choice of deposition temperature was arrived at by keeping in mind the melting point of Al being 660.32°C. This could ensure abundant Al vapors during the deposition process. So, the need was to maintain the temperature of the furnace just above melting point of both Zn and Al. As the furnace temperature reached the set value, high purity O_2_ and Ar in the ratio of 20:80 was introduced into the quartz tube. Flow rate of O_2_ was maintained at 200 sccm (standard cubic centimeters per second). The purity of O_2_ and Ar were 99.8% and 99.999%, respectively. The duration of heating was maintained at 120 min for all samples based on the preliminary results. The flow rate of reactant gas (O_2_) and carrier gas (Ar), duration of growth time, growth temperature, and pressure were carefully set and monitored since slightest change in these parameters may affect the result of the samples obtained. Exactly at the end of 120 min of heating, the flow of reactant and carrier gases were stopped and the furnace was set to cool down to room temperature before removing the sample. Once the furnace got cooled to near room temperature, the sample was removed from it. Grayish white deposits were observed on the silicon substrate. The same procedure was repeated for all samples of different dopant concentrations.

### Doping mechanism of ZnO:Al

Due to their confined electronic states to a very small volume in nanocrystals, doping leads to phenomena not found in the bulk counterparts. Although the underlying mechanism responsible for these observations are still under investigation, we believe that the following reactions spontaneously occur during the deposition of ZnO:Al NSs.

(2.1) $$2\;\mathrm{Al}+3\;\mathrm{ZnO}\to +3\;\mathrm{Zn}+3\;V_{o}$$

(2.2) $$2\;\mathrm{Al}+3\;\mathrm{ZnO}\to +3\;{\mathrm{Zn}}_{i}$$

It is expected that doubly charged donors including oxygen vacancies (*V*_o_) and zinc interstitials (Zn_*i*_) would be formed by the extrinsic doping of Al. This is possible if the incorporated Al atoms take oxygen from ZnO and form either or inside the ZnO matrix. As the standard Gibbs-free energy change of these reactions is largely negative (-618 kJ mole^-1^) [3], it is believed that the formation of m^*^/*m*^o^ is responsible for the extrinsic doping of ZnO:Al, which is contrary to the conventional doping mechanism based on the substitution of foreign elements. Doping takes place by incorporating Al atoms in which charged donors would be formed at or near the Al_2_O_3_/ZnO interface in compensation for free electrons. The electrons around these donors could be localized within the Bohr radius (a_H_) of ZnO as stated below:

(2.3) $$a_{h}=\frac{a}{m^{*}m}$$

where *a*_o_ = Bohr radius of H atom (0.53 Å), *ϵ*_r_ = relative permittivity of ZnO (81), m^*^ = effective mass of an electron in ZnO (0.318), *m*_o_ = mass of an electron, and *a*_H_ = Bohr radius of ZnO.

Theory in reference [3] suggests that of ZnO in Equation (2.3) is approximately 14 Å. Since donated electrons orbit around charged donor with the radius, the repulsion force between electrons belonging to adjacent donors could suppress the donation of additional electrons. The Coulomb repulsion force between adjacent charged donors may also cause decrease of carrier concentration in the same manner. Thus, these repulsion forces could cause the effective field for doping around each donor. These effective fields probably limit the doping efficiency of Al atoms within a single Al_2_O_3_ layer.

### Alloying evaporation method

**Figure 2:**
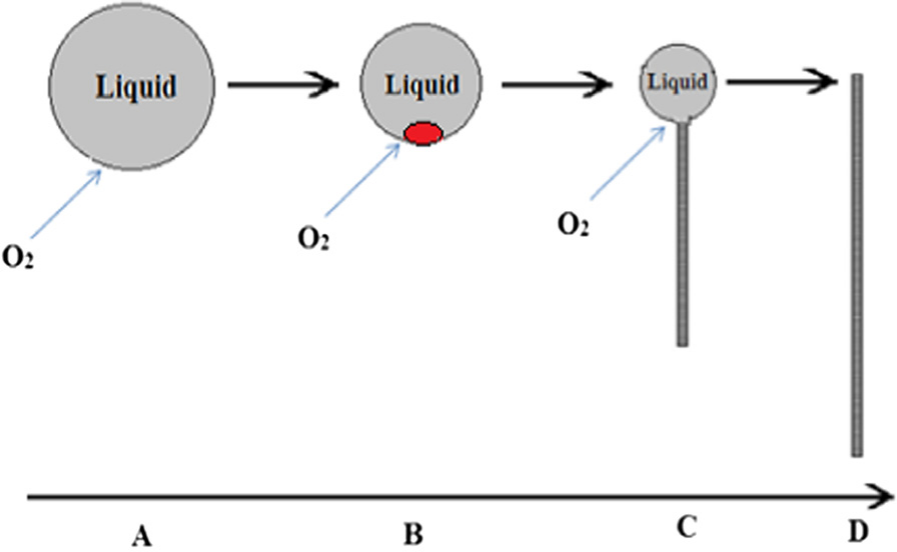
The self-catalytic model of ZnO:Al growth.

According to the self-catalytic growth mechanism proposed by Dang et al. [4], the process completes in four major steps. Figure 2 best explains the particular growth mechanism. It can be understood as follows: (A) As soon as the temperature of the furnace reaches the melting point of the Zn powder, it starts to melt and form a large quantity of melting liquid drops of size approximately identical to those of the original solid metal particles. (B) The oxygen in the inlet flow is then absorbed by the melting metal at some points on the surface resulting in some metal oxide nanoclusters, which serve as nuclei for the nanowire growth. (C) Depending on the availability of source metal reactants and appropriate quantities of O_2_, the growth of metal oxide NWs begins and continues after the formation of the nuclei. (D) Growth of ZnO NWs terminates when the source metal is exhausted.

The atomic ratio of Zn:O on the tip and root of a NR was not the same. Concentration of oxygen on the tip of the ZnO NRs exceeded the root [5]. The fact is attributed to the alloying of Al/Zn mixed sources during the growth of NRs. The Al vapor pressure is much lower than that of Zn at the same temperature range. However, Zn and Al sources in the process would form a certain quantity of Zn-Al alloy by interdiffusion through the Zn/Al interface. Since the bond energy of Zn-Al, 0.101 eV, is higher than that of Zn-Zn, 0.054 eV, which may cause the decreasing of Zn vapor pressure in the quartz tube with the alloying of Zn and Al during the deposition process. On the other hand, the flow rate of oxygen in the furnace is constant. As a result, the tip of ZnO NRs exhibits lower zinc concentration than the root. This particular process has contributed to unique optical properties of the NRs as described below. With higher zinc and lower oxygen concentration at the root of NRs, it exhibits green emission that is attributed to the existence of oxygen vacancy.

## Results and discussion

### Synthesis ZnO:Al nanowires

**Figure 3:**
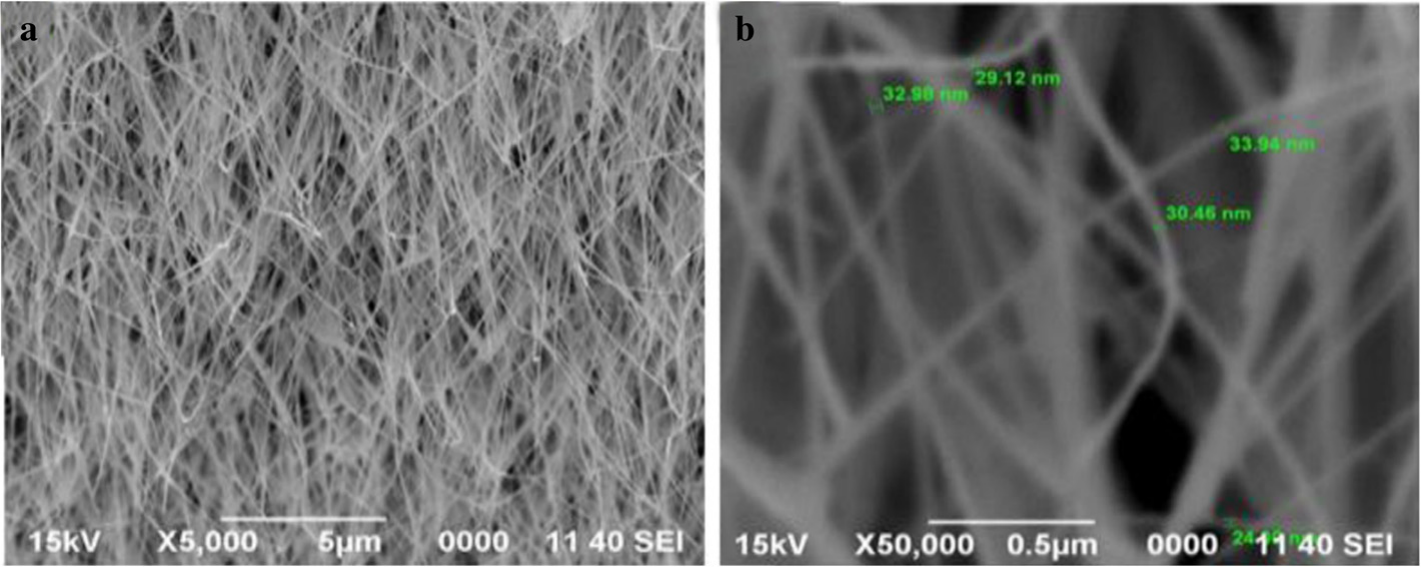
**SEM images of randomly oriented mesh of ZnO nanowires synthesized on silicon substrates. (a)** Low magnification and **(b)** high magnification.

The experimental results of ZnO:Al NRs grown from alloying evaporation deposition (AED) growth mechanism using thermal evaporation technique are illustrated. The growth parameters such as growth temperature, growth duration, deposition pressure, flow rate of oxygen gas, and type of substrate have a huge effect on the formation of NSs. However, we have narrowed down and focused our study on the effects of dopant concentrations keeping the rest of the parameters invariant. So, accordingly, the characterization analysis for structural and optical properties and explanations thereof are recorded in the following. Data obtained from various samples with different dopant concentrations were analyzed using XRD, scanning electron microscopy (SEM), field emission scanning electron microscopy (FESEM), energy-dispersive analysis X-ray (EDAX), and photoluminescence (PL) and the results are interpreted in the following subtopics.SEM images also confirmed the formation and existence of ZnO NWs. Figure 3 is the result of ZnO nanowires grown for 120 min at 700°C with 200 sccm flow rate of oxygen gas. A bushy mesh of NWs can be observed in Figure 3a. On an average, the NWs are approximately 30 nm in diameter and several microns in length as can be known from Figure 3b. It is of immense assurance that the experimental setup is impressive and capable of forming NWs.

### Structural properties of undoped ZnO nanowires

**Figure 4:**
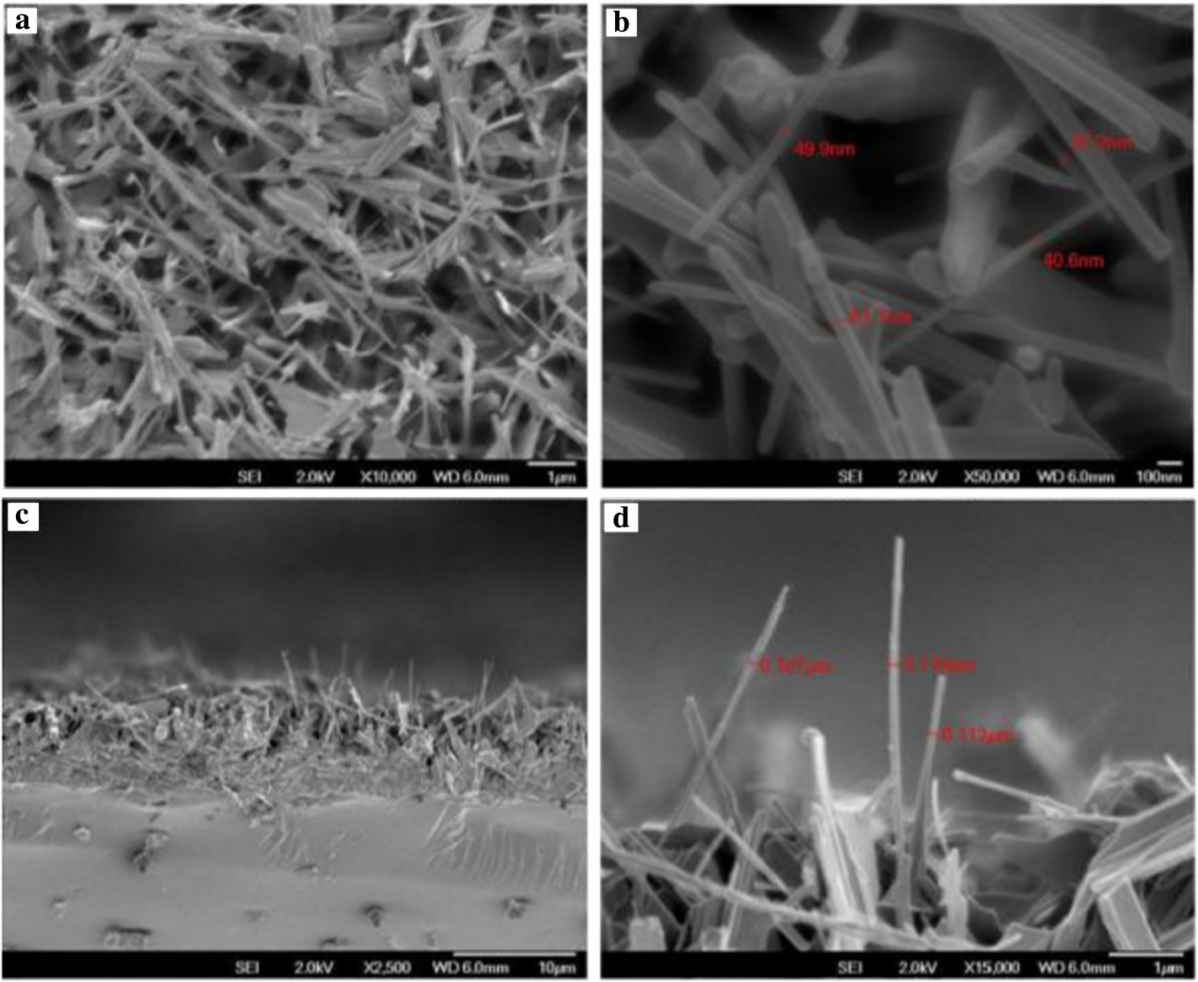
**FESEM images of undoped ZnO nanowires synthesized on Si substrate. (a, b)** Surface view, **(a)** low magnification, and **(b)** high magnification. **(c, d)** Cross- sectional view, **(c)** low magnification and **(d)** high magnification.

**Figure 5:**
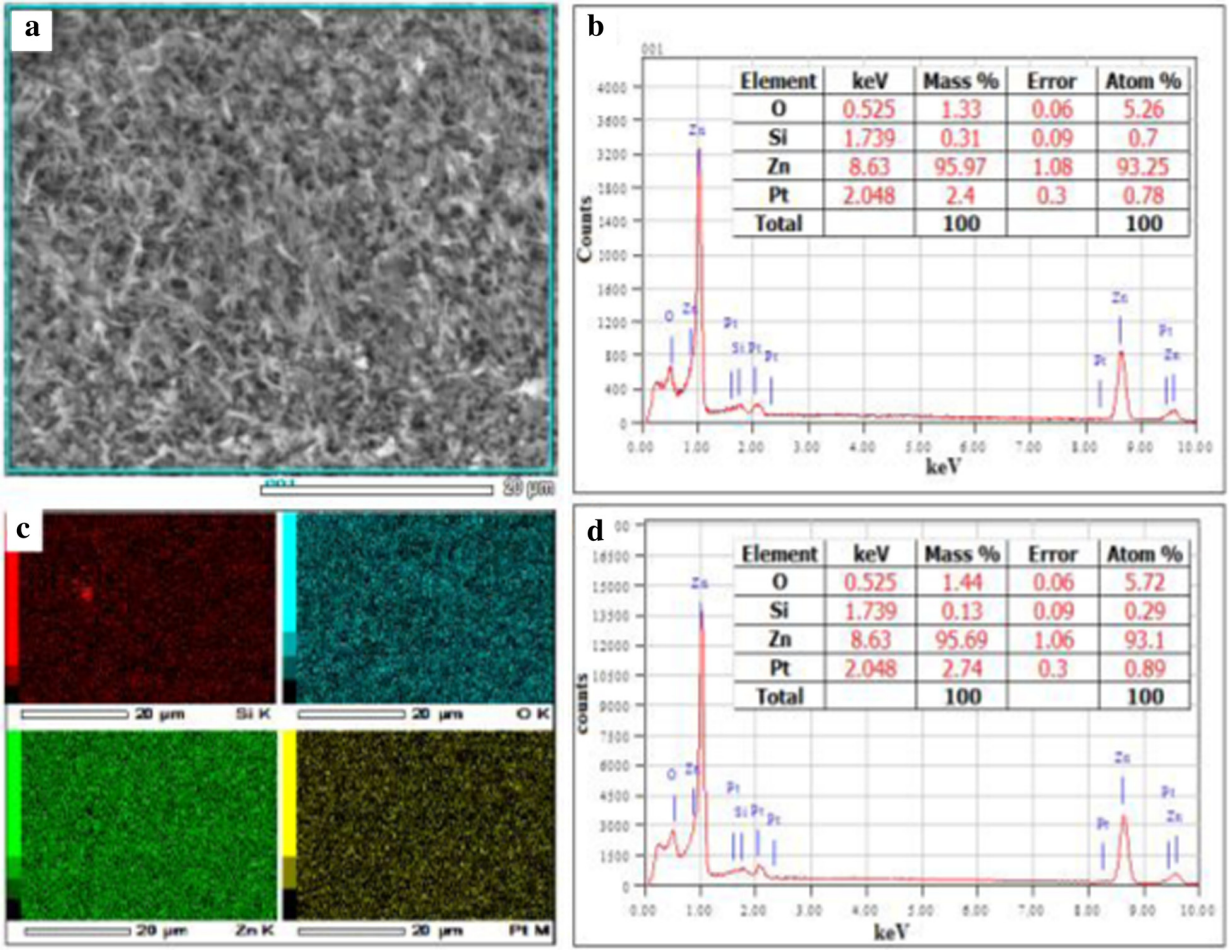
**Detection position of EDAX spectra and image of element mapping. (a, b)** Detection position of EDAX spectra of the ZnO nanowires sample and its respective EDAX specta. **(c, d)** Image of element mapping of the sample and its EDAX spectra.

The FESEM images in Figure 4 indicate that ZnO NWs are randomly oriented and of very high density. Figure 4 shows the nanowire grown with 120 min at 700°C with 200 sccm flow rate of oxygen gas. The NWs have a high aspect ratio with varying diameter of approximately 30 to 60 nm and length extending several microns as can be noticed in Figure 4b. It can be established that this simple method is a viable method of ZnO NWs synthesis. From Figure 4d, some of the NWs are vertical while many are tilted or slanted and are also having varying lengths. We can also observe in cross-sectional image in Figure 4c,d that the NWs are packed at the bottom in comparison with the surface where we can see lesser number of NWs sprouting out of the thickness.To determine the purity and composition of the sample, energy-dispersive analysis X-ray (EDAX) analysis was carried out. The result indicates that ZnO NWs obtained are of high purity. In Figure 5b, the EDAX spectra shows that sample consists of exclusively Zn = 93.25 at.% and O = 5.26 at.%. The presence of platinum (Pt) in trace is as a result of coating sample with Pt while preparing for FESEM analysis for which EDAX is attached with. Trace of Si detected is also accounted from the substrate. So, considering the detection of elements in the sample, it can be very well considered to have obtained high purity ZnO NWs.The sample mapping in Figure 5c shows that the elements are distributed evenly in the sample where density of O = 5.72 at.%, Si = 0.29 at.%, Zn = 93.10 at.%, and Pt = 0.89 at.% as shown in the form of image in sequence of elements presented. Inset in Figure 5d shows the composition and distribution data of the sample mapping.

### Effect of dopant concentration on ZnO:Al nanostructure

**Figure 6:**
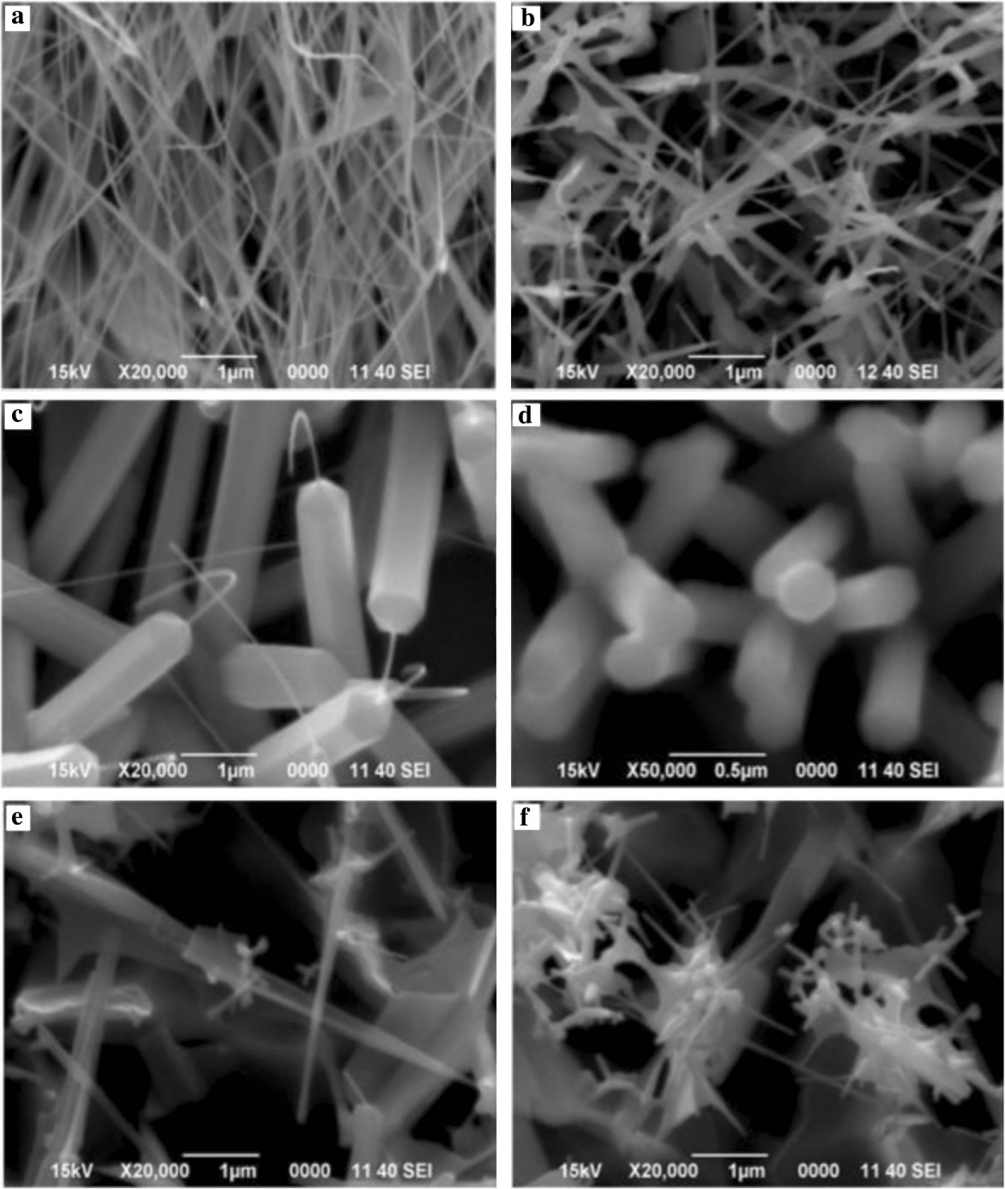
**Comparative SEM images of undoped and Al doped ZnO nanowires. (a)** 0 at.% Al (undoped), **(b)** 0.6 at.% Al, **(c)** 1.2 at.% Al, **(d)** 2.4 at.% Al, **(e)** 4.7 at.% Al, and **(f)** 11.3 at.% Al.

The values of dopant concentrations were between the ranges of 0 at.% to 11.3 at.% as shown in Table 1.It is obvious that the varying dopant concentrations have a profound impact on the structural properties of NWs. A clear comparison can be made in terms of the structural properties of ZnO:Al from Figure 6. In the case of 0.6 at.% Al dopant concentration in Figure 6b, there has been not much impact as the dopant concentration is relatively small. So, the NSs look almost comparable to undoped as in Figure 6a except that the width of the NSs has grown bit larger. But as the concentration increases to 1.2 at.% as in Figure 6c, it has brought about a remarkable change in the structural properties. Hexagonal-shaped NSs are formed which extends to a length of few microns and then narrows like sharpening the pencil and ultimately leads to an elongated core which appears like an exposed core of pencil. At a glance, having an interesting tail for every structure can be observed. The tails look flexible since some are bent like hook while others look slightly bent only. Actually, the NSs are in the process of forming a well-defined shape. It is very likely that the dopant concentration was less than required for the formation of well-defined hexagonal shape. However, the shape itself appears thought-provoking and invites lots of curiosity and zeal for further investigation.Viewing image Figure 6d, it can be well established that a perfect hexagonal NSs looking ‘pencil-like’ have been formed. It can be considered that 2.4 at.% were the possible optimum dopant concentration for the synthesis of the NS. The randomly oriented NS appear well formed and near uniform in size and length. From the EDX analysis, it can be confirmed that Al has been doped into the structure. EDX result shows that 0.08 at.% Al is present in the NS synthesized which can be known from Figure 6b. The sample mapping also indicates that 0.13 at.% Al is present in the sample. To the best of our knowledge, no previous results exhibit such morphology fabricated by thermal evaporation method.

**Table 1:** **Varying dopant concentrations at constant temperature, growth time, and flow rate of O**_**2**_

**Number**	**Growth time (min)**	**Growth temperature (°C)**	**Flow rate of O**_**2 **_**gas (sccm)**	**Dopant concentration (at.%)**
1	120	700	200	0
2	120	700	200	0.6
3	120	700	200	1.2
4	120	700	200	2.4
5	120	700	200	4.7
6	120	700	200	11.3

**Figure 7:**
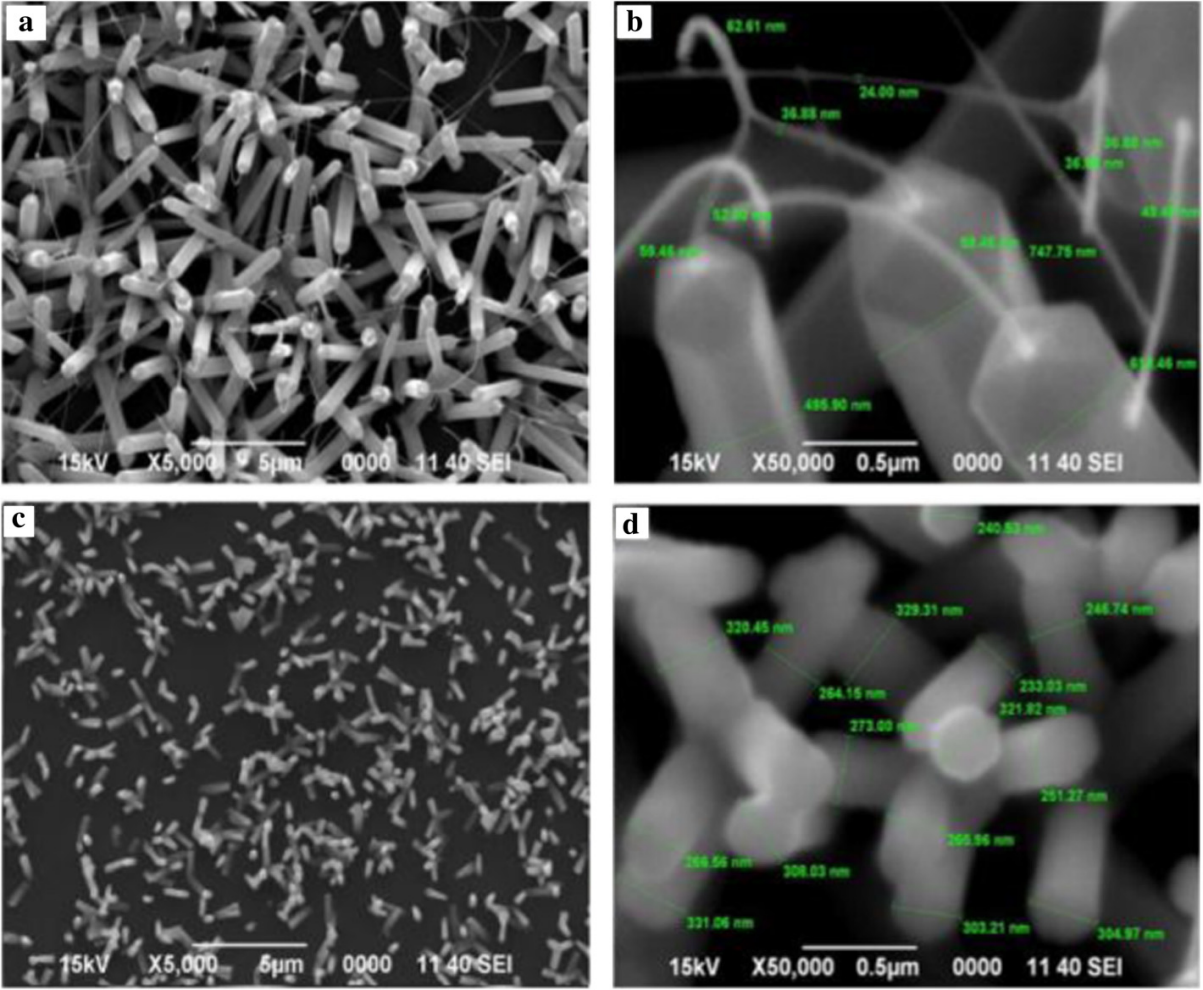
**SEM images of Al-doped ZnO nanowires. (a, b)** 1.2 at.% Al, low and high magnification. **(c, d)** 2.4 at.% Al, low and high magnification.

**Figure 8:**
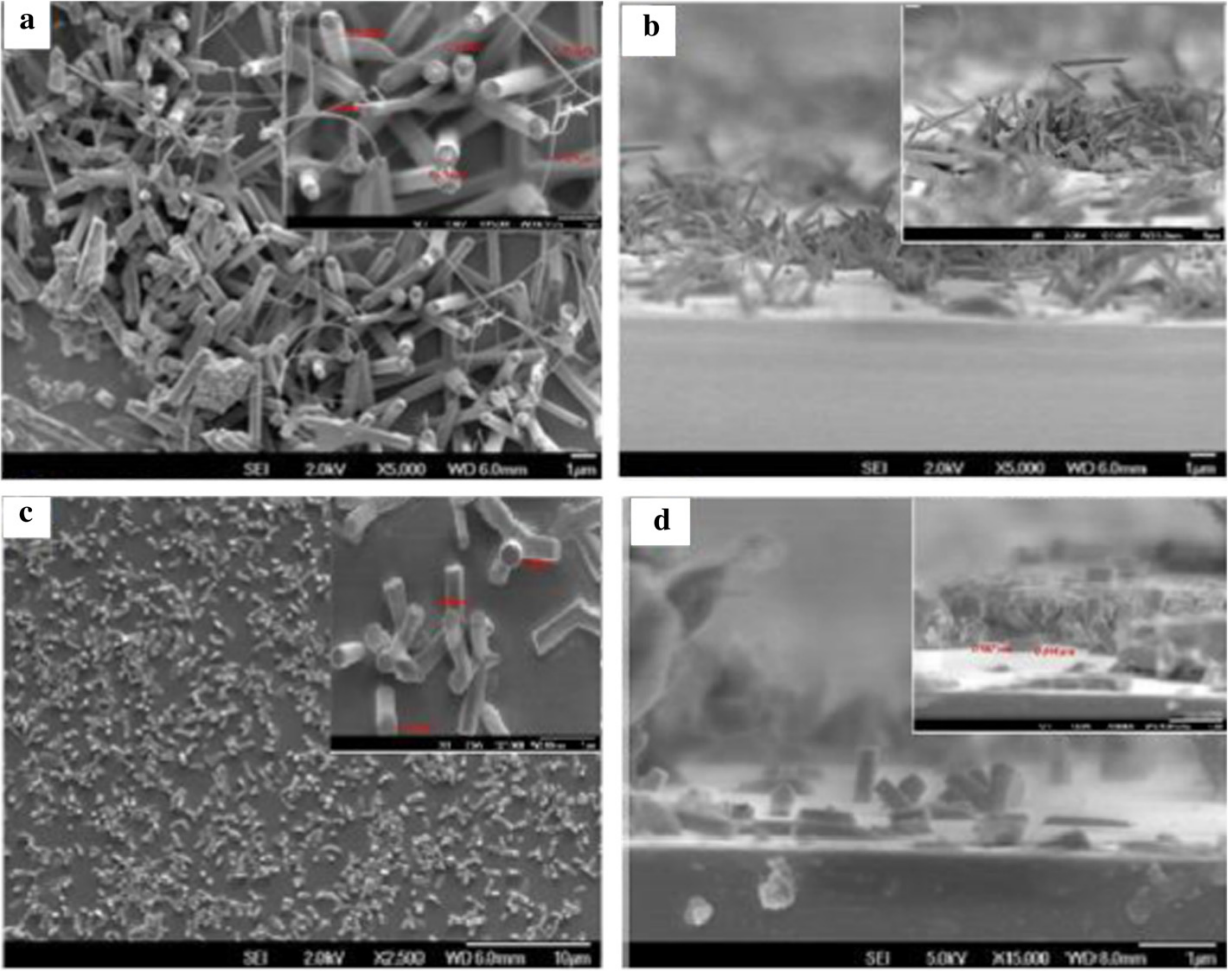
**FESEM images of Al doped ZnO nanowires. (a, b)** 1.2 at.%, (a) surface view with inset showing high magnification and **(b)** cross-sectional view with inset showing high magnification. **(c, d)** 2.4 at.%, **(c)** surface view with inset showing high magnification and **(d)** cross-sectional view with inset showing high magnification.

**Figure 9:**
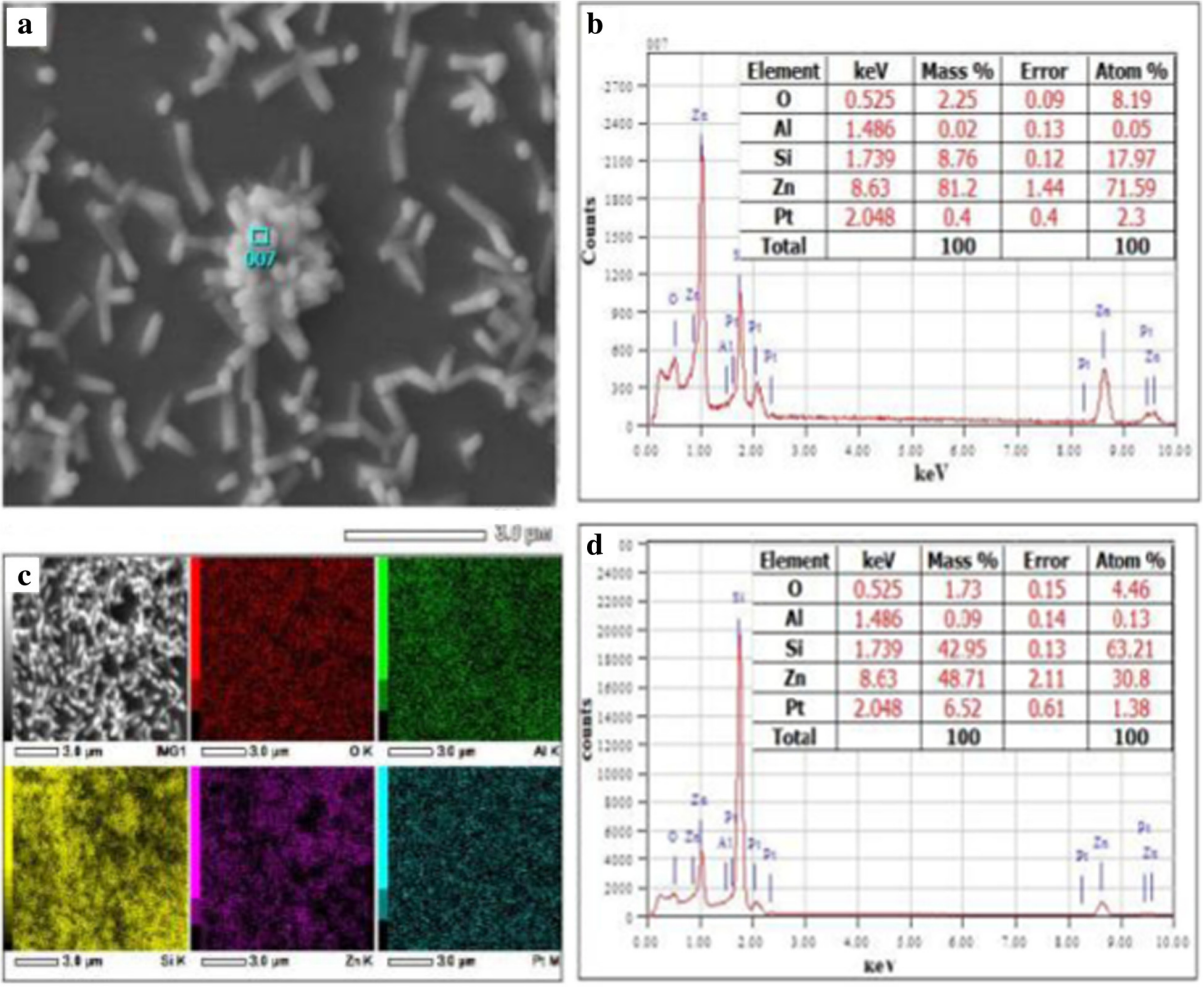
**Detection position of EDAX spectra of 2.4 at.% Al-doped ZnO:Al nanowires and image element mapping. (a, b)** Detection position of EDAX spectra of 2.4 at.% Al-doped ZnO:Al nanowires sample and its respective EDAX spectra. **(c, d)** Image of element mapping of the sample and its EDAX spectra.

When the dopant concentration exceeds beyond 2.4 at.%, the perfect hexagonal shape of the NS are lost. It appears cylindrical in shape with a needle-like extensions from the tips of NRs. The base of NRs appears larger than the tip although at a constant temperature which otherwise if the reaction temperature was raised, the nanowires became thicker because of the enhanced lateral growth [6]. Along with, undefined structures appear in which some look spiky and thorny and others may be nanosheets as in Figure 6e,f which corresponds to 4.7 at.% and 11.3 at.% dopant concentrations, respectively. In the work of Chen et al. [7], further introduction of more Al ions (6 at.%), they obtained network-like nanosheets rather than tubes and rods which was the case for lower dopant concentrations. It is noticeable that beyond 2.4 at.% dopant concentration, it does not contribute to good structural properties of ZnO:Al NWs. We are not very sure if such structures with spiky shapes will have any practical use in any field. ZnO NSs doped with 3 at.% Al hydrothermally treated for different times were found to be NRs companying with the NTs [7]. Lin et al. [8] argues that the aluminum doping concentration can be controlled simply by adjusting the distance between the substrates and source materials. However, since substrate is vertically placed above the source, there is no scope to change this parameter.From Figures 7 and 8, the Al-doped ZnO nanowires images are well established. The SEM images in Figure 7 tell us the optimum dopant concentration, a well-defined nanowires are formed and its hexagonal shaped can clearly be seen. When the dopant concentration is increased to 2.4 at.%, it is depleted vigorously making rise to development of tail which entangled from top of the nanowires. FESEM images in Figure 8 are purposely provided to give much clearer images of Al-doped ZnO nanowires with similar growth condition as that of the nanowires in Figure 7.While in Figure 9, EDAX spectra proved the existence of Al as dopant in the respective set of experiment where a significant rise of Al spectrum is showed. For better understanding, an inset showing element mapping of the sample alongside the EDAX spectra of the mapping with inset showing element composition in mass and atomic percentage.

**Figure 10:**
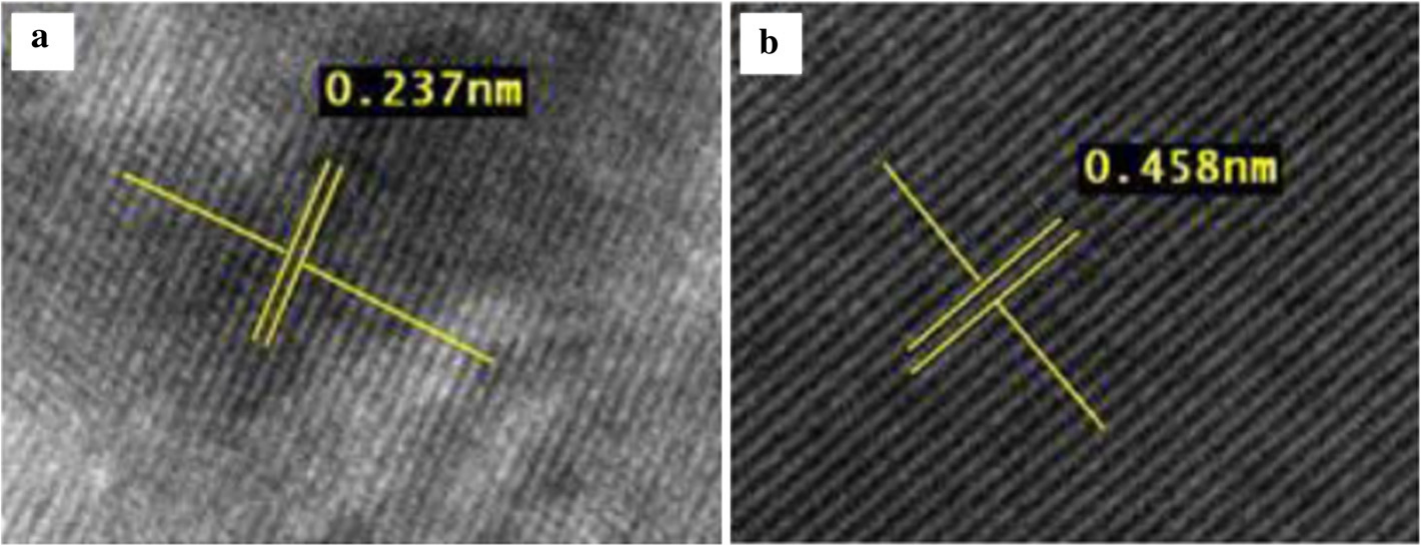
**HRTEM images of (a) ZnO and (b) ZnO:Al nanowires.** Showing the lattice spacing of 0.24 nm and 0.46 nm, respectively.

The HRTEM image of a single ZnO nanowire is shown in Figure 10. It can be seen clearly that the ZnO crystal lattice is well-oriented with no observable structural defects over the whole region. This result is comparable to those obtained by the earlier works [9,10]. The lattice spacing of the ZnO and ZnO:Al nanowire are about 0.26 and 0.46 nm, respectively corresponding to the distance between two (002) crystal planes, confirming that the ZnO nanowires are referentially grown along the [001] direction. Figure 10a shows the undoped ZnO nanowires, and Figure 10b shows doped ZnO nanowires, ZnO:Al which both is grown with 2.4 at.% Al dopant concentration at 700°C and deposited for 120 min.

### Effect of dopant on ZnO:Al optical properties

PL spectra were recorded at room temperature using Luminescence LS55 model-Perkin Elmer (Waltham, MA, USA) at the Physic Department, Universiti Teknologi Malaysia.

**Figure 11:**
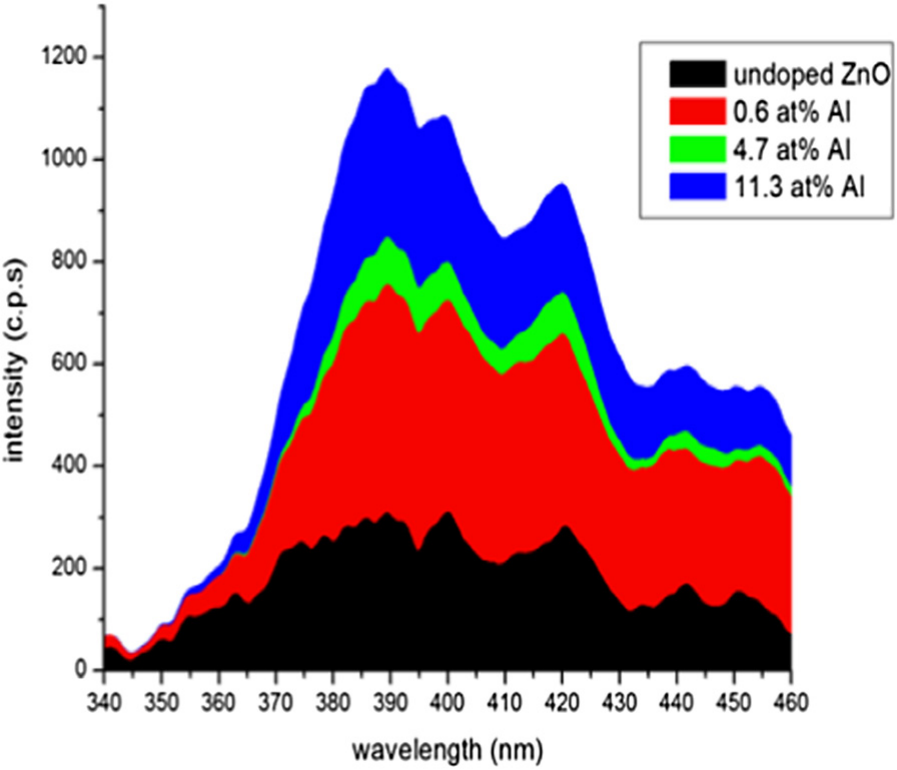
PL spectra of the as-synthesized ZnO:Al nanowires on silicon substrate showing intensity versus wavelength.

According to Figure 11, strong ultraviolet (UV) emission band located at approximately 389 nm (*E*_g_ = 3.19 eV) for undoped as well as for all doped ZnO:Al NWs can be seen which agrees with the PL spectra reported in literature [9]. For the same substrate used in [10], only strong peaks corresponding to UV emissions were observed, whereas in the present work besides the strong UV emission peak, multiple other low intensity peaks appear. The peaks correspond to the following wavelengths: 400 nm (*E*_g_ = 3.1 eV), 420 nm (*E*_g_ = 2.95 eV), 442 nm (*E*_g_ = 2.81 eV), and 452 nm (*E*_g_ = 2.74 eV). It is believed that the oxygen vacancies were located in the interfacial region of the ZnO NWs which have contributed to the emission of those peaks.

The peaks appear nearly identical in shape for all samples except that they differ in the intensity only. The intensity of the peaks increases and become sharper as the dopant concentration increase. For undoped, UV emission peaks are slightly broader whereas the peaks are narrower and sharper and of higher intensity for all doped samples and become sharper as the dopant concentrations increase. From here, we know that the optical properties of nanostructures also differ with the aspect ratio of the nanostructure in which we observe only UV emission for low aspect ratio and vice versa. The increase in peak intensity with the corresponding increase in dopant concentration can be attributed to near band-edge emission from crystalline ZnO and recombination of free excitons. This is in good agreement with the findings reported in [11].

**Figure 12:**
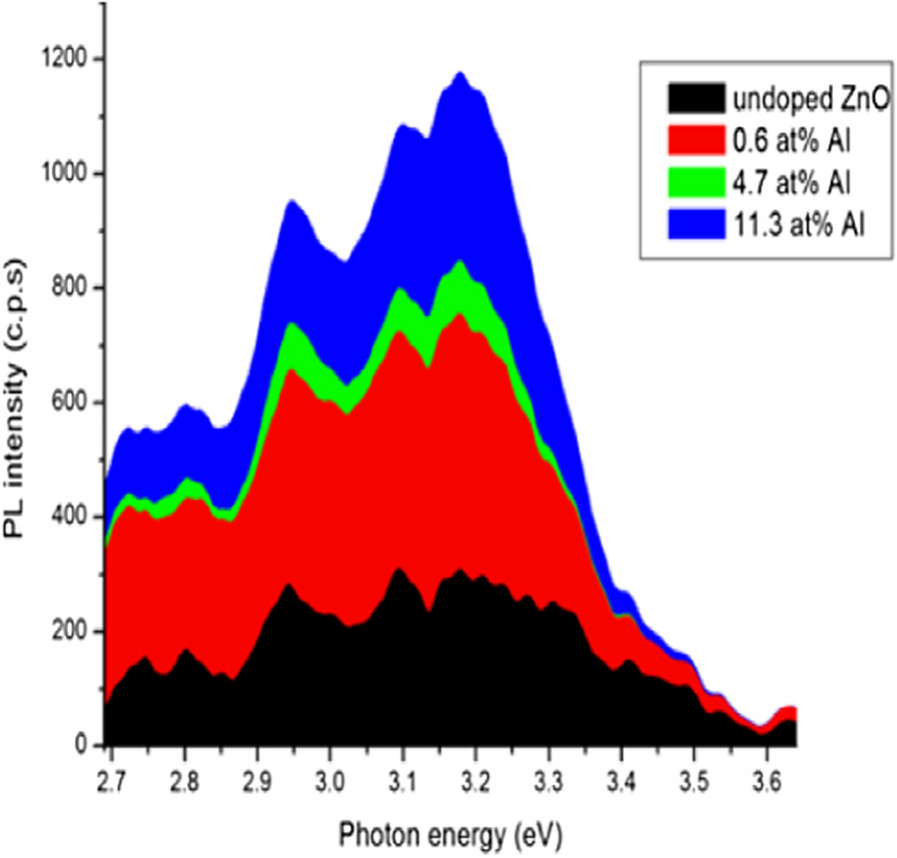
PL spectra of the as-synthesized ZnO:Al nanowires on a silicon substrate showing intensity versus energy.

In addition to the UV emission, broad oxygen vacancy-related emission band centered at the following energy band gaps (*E*_g_ = 3.1 eV), (*E*_g_ = 2.95 eV), (*E*_g_ = 2.81 eV), and (*E*_g_ = 2.74 eV) can be observed for all doped ZnO:Al NRs as can be observed in Figure 12. The peaks correspond to a range between violets and blue (lower visible spectrum). These relatively weak near-band edge emission and significant defect-related emission property of these nanowires are believed to be beneficial to their photocatalytic activity [6]. It is understood that surface oxygen deficiencies are electron capture centers, which can reduce the recombination rate of electrons and holes. The emissions in visible range is known to originate from the oxygen vacancies and Zn interstitials produced by the transition of excited optical centers from the deep to the valence level. The emission band at 420 nm is strongest in the 11.3% Al-doped ZnO that can be attributed to the high level of structural defects (oxygen vacancies and zinc interstitials and/or presence of Al ions replaced with Zn ions) in the ZnO lattice structure, which manifest as deep energy levels in the band gap [6]. Thus, the intensity of undoped and doped PL spectra corroborates the enhanced defect levels in the specimens, with a corresponding increase in the concentration of Al.

It is obvious that well-doped ZnO nanostructures have been obtained especially sample ZnO:Al 4 which was doped with 2.4 at.% Al. From the EDAX result, it is very well confirmed that Al was incorporated into the NSs. In fact, the NRs contained 0.05 at.% Al as can be known from the Figure 9b inset table. During the doping process, rather than of Zn atoms being substituted by Al atoms, we believe that oxygen vacancies (*V*_o_) and zinc interstitials (Zn_*i*_) were formed as Al atoms combined with oxygen in ZnO. Indeed, it was a deviation from the conventional doping mechanisms in which Al is thought to substitute Zn atoms. Our idea is well supported by the PL spectra in Figure 12 in which emissions peaks in visible range can be attributed to formation of oxygen vacancies and zinc interstitials which also agrees with reference [6].

## Conclusions

Dopant plays an important role on controlling the morphology of ZnO NWs. As evident from the result, it indicates that the optimum dopant concentration to be about 2.4 at.% where a ‘pencil-like’ hexagonal NSs were formed. We also obtained very interesting NSs at 1.2 at.% which appear pencil-like but having a tail. We assume 2.4 at.% to be an optimum dopant concentration necessary which resulted in the formation of defined hexagonal shaped pencil-like NSs. Once again, we would like to stress on the proposed method to obtain Al-doped ZnO (ZnO:Al) NSs. The intensity of UV emission increases with increase in doping which is observed on the PL spectra presented before. Especially, the UV emission is enhanced which is an indication of its practicality in optical sensing application. From SEM, FESEM, and PL images, we felt that the doping mechanism occurs via formation of oxygen vacancies (*V*_o_) and zinc interstitials (Zn_*i*_) rather than substitution as is the case for conventional methods.

## Competing interests

The authors declare that they have no competing interests.

## Authors' contributions

SS (Suhaimi) designed and performed the experiments, participated in the characterization and data analysis of SEM, FESEM, HRTEM and PL, and prepared the manuscript. TD participated in the SEM, FESEM, and PL characterization. SS (Sakrani) and AKI participated in the revision of manuscript. SS (Sakrani) participated in the monitoring of the experimental work, data analysis, and discussion of the manuscript. All authors read and approved the final manuscript.
